# Establishing specialized health services for professional consultation in euthanasia: experiences in the Netherlands and Belgium

**DOI:** 10.1186/1472-6963-9-220

**Published:** 2009-12-04

**Authors:** Yanna Van Wesemael, Joachim Cohen, Bregje D Onwuteaka-Philipsen, Johan Bilsen, Luc Deliens

**Affiliations:** 1 End-of-Life Care Research Group, Vrije Universiteit Brussel, Brussels, Belgium; 2 VU University Medical Centre, Department of Public and Occupational Health, EMGO Institute for Health and Care Research, Expertise Centre for Palliative Care, Amsterdam, the Netherlands

## Abstract

**Background:**

The Netherlands, Belgium, and Luxembourg have adopted laws decriminalizing euthanasia under strict conditions of prudent practice. These laws stipulate, among other things, that the attending physician should consult an independent colleague to judge whether the substantive criteria of due care have been met. In this context initiatives were taken in the Netherlands and Belgium to establish specialized services providing such consultants: Support and Consultation for Euthanasia in the Netherlands (SCEN) and Life End Information Forum (LEIF) in Belgium. The aim of this study is to describe and compare these initiatives.

**Methods:**

We studied and compared relevant documents concerning the Dutch and Belgian consultation service (e.g. articles of bye-laws, inventories of activities, training books, consultation protocols).

**Results:**

In both countries, the consultation services are delivered by trained physicians who can be consulted in cases of a request for euthanasia and who offer support and information to attending physicians. The context in which the two organisations were founded, as well as the way they are organised and regulated, is different in each country. By providing information on all end-of-life care matters, the Belgian LEIF seems to have a broader consultation role than the Dutch SCEN. SCEN on the other hand has a longer history, is more regulated and organised on a larger scale and receives more government funding than LEIF. The number of training hours for physicians is equal. However, SCEN-training puts more emphasis on the consultation report, whereas LEIF-training primarily emphasizes the ethical framework of end-of-life decisions.

**Conclusion:**

In case of a request for euthanasia, in the Netherlands as well as in Belgium similar consultation services by independent qualified physicians have been developed. In countries where legalising physician-assisted death is being contemplated, the development of such a consultation provision could also be considered in order to safeguard the practice of euthanasia (as it can provide safeguards to adequate performance of euthanasia and assisted suicide).

## Background

While physician-assisted suicide is regulated in Oregon, Washington, the Netherlands, and Luxembourg and is legally performed in Switzerland since 1990 [1-3], there are only three countries in the world where euthanasia is legal: the Netherlands and Belgium adopted a law in 2002 [4,5] and Luxembourg became the third country to do so on March 16^th ^2009 [6]. All three laws stipulate substantive and procedural criteria that must be met for euthanasia to be legally performed. The substantive criteria require, among other things, that the patient's request must be voluntary, well-considered, repeated, and not the result of any external pressure; that the patient must be in a medically futile state of constant and unbearable physical or psychological suffering which cannot be alleviated, and is the result of a serious and incurable condition caused by illness or accident; that the physician must fully inform the patient about his/her health condition and prospects (diagnosis and prognosis), and that physician and patient must arrive at the conclusion that there is no reasonable prospect of improvement in the patient's situation. The procedural criteria consist of mandatory notification of the euthanasia case to the official review committee [7], and consultation of a colleague by the attending physician, hereafter called the consultant, who is independent or impartial from both the patient and the attending physician [4,5], and competent to judge the patient's condition. This consultant must read the medical file and examine the patient in order to judge whether the substantive criteria have been met, i.e. judge the serious and incurable nature of the condition, ascertain that the patient's physical or psychological suffering is constant, unbearable, and without prospect of improvement, and that the patient's request was voluntary, well-considered, and repeated (in Belgium and Luxembourg the law only prescribes this in patients not expected to die in the near future). The Dutch law also stipulates that the consultant should conclude that there are no reasonable alternatives [5]. The consultant must make a written report regarding his or her conclusions.

Consultation in the case of a euthanasia request, as defined by the laws on euthanasia, is very different from an informal discussion between physicians which might occur in other kinds of end-of-life decision-making. Given the seriousness and irreversibility of euthanasia, the consultant has to determine whether the substantive legal requirements of due care are met, and the judgment of the attending physician was made with due care. The consultation of a second physician in euthanasia requests is intended to build a control mechanism into the procedure and prevent unwarranted euthanasia cases. It is also intended to monitor and safeguard the quality of the practice of euthanasia.

The laws in all three countries stipulate that the consulted physician must be independent, impartial and competent to judge the pathology of the patient. However, the consultant is also expected to judge aspects such as existential suffering and feelings of hopelessness, which are more inherent to the final stage of life than to the patient's pathology [8-10]. Additional skills therefore seem warranted for a consultant. Ideally, the consultant is someone who does not a priori object to euthanasia, and has a certain amount of experience with or knowledge of end-of-life care and/or euthanasia. Finding such an independent consultant may be difficult for a physician confronted with a euthanasia request. In this context initiatives were taken, in the Netherlands and in Belgium, to establish specialized services to provide such consultants. While the Dutch and the Belgian laws [11] and the notification procedures of euthanasia in both countries have been extensively described and compared elsewhere [7], no studies have described the function and functioning of these specialized consultant health services within the context of a law on euthanasia. This paper aims to describe such specialized health services as established in the Netherlands ('Support and Consultation on Euthanasia in the Netherlands', i.e. SCEN) [12] and in Belgium ('Life End Information Forum', i.e. LEIF) [13]. LEIF is a Flemish initiative and hence in principle only available in the Dutch-speaking part of Belgium; in the French-speaking part a similar, albeit less elaborate initiative has been developed.

The SCEN and LEIF projects will be described and compared in terms of their development, aims, tasks, functioning and organisation.

## Methods

We studied and compared relevant documents concerning SCEN and LEIF. To obtain an overview of the development, aims, tasks, functioning and organisation of SCEN, the evaluation report about the implementation and effects of SCEN was studied [14] as well as the results of the annual written inventory of activities of SCEN physicians from 2004 to 2006 [15-17]. Additionally, the training book for SCEN physicians [18], the checklist used by SCEN to draw up the consultation report, the protocol used as a guideline for the consultation procedure [19] and the website of the Royal Dutch Medical Association [20] were explored as information sources. Information on the Life End Information Forum in Flanders was acquired through the LEIF website [21], the bye-laws, the LEIF magazine [22], publications concerning LEIF [13,23,24] and the training folders the physicians receive while undergoing training. Furthermore, because there is relatively less written information available about LEIF than about SCEN, an open interview was conducted with the director and training moderator of LEIF to complement the collected information. The persons consulted for information about SCEN and LEIF were notified that their contribution would be used for a comparing paper and they consented to this.

## Results

### Development

SCEN and LEIF were established in differing contexts (Table 1). In the Netherlands, where euthanasia had already been taking place without prosecution for more than a decade [8,25], the Royal Dutch Medical Association and the Association of General Practitioners wanted to professionalize the consultation process and thus make physicians take responsibility for the quality of the practice. They initiated a pilot project in Amsterdam (Support and Consultation on Euthanasia in Amsterdam, SCEA) in 1997 and extended it to the rest of the country in 1999, after an evaluation of its implementation [25]. In Belgium, the legalisation process of euthanasia was much shorter and enjoyed less support from associations of health care professionals [26]. LEIF was established in February 2003, after the euthanasia law had come into effect, by individual professionals with experience in palliative care and by the association 'Right to Die with Dignity'. Their aim was twofold: to create a service that could refer people to the right health care professionals in end-of-life matters, and to increase physicians' knowledge about palliative care and euthanasia through training programs.

**Table 1:** development and foundation of LEIF and SCEN

Development & Foundation	LEIF	SCEN
Initiators	Initiative of individuals with experience in end-of-life care and the pluralistic association 'Right to Die with Dignity'*	Initiative of the Royal Dutch Medical Association and the Association of General Practitioners †
		
Year of founding	In 2003, 6 months after the euthanasia law *	In 1997, before the euthanasia law ‡
Covering region	Provided for the 6 provinces in Flanders*	First a pilot project in Amsterdam (SCEA) in 1997, since 1999 in the rest of the country ‡

* Source: Interview with the director and training moderator of LEIF and Distelmans W: **LEIF: het LevensEinde InformatieForum (LEIF: the Life End Information Forum)**. *Neuron *2008, **13 (3)**:144-146.

† Source: http://knmg.artsennet.nl

‡ Source: Onwuteaka-Philipsen B, van der Wal G: Support and consultation for general practitioners concerning euthanasia: the SCEA project. *Health Policy *2001, 56:33-48.

Jansen-van der Weide M, Onwuteaka-Philipsen B, van der Wal G: Implementation of the project 'Support and Consultation on Euthanasia in The Netherlands' (SCEN). *Health Policy *2004, 69:365-373.

### Aims and tasks

SCEN and LEIF were both initially developed to provide independent and competent second physicians as consultants in euthanasia requests, as required by law (Table 2); these physicians are however also able to provide information and support concerning euthanasia outside the context of consultation. The scope of LEIF is broader than that of SCEN, as its aim is also to provide consultation in other end-of-life decisions, including palliative care, to other physicians as well as to patients, and to provide the wider public with information about euthanasia and other end-of-life matters.

**Table 2:** aims and tasks of LEIF and SCEN

Tasks	LEIF	SCEN
**Tasks**	* Provide information and support about euthanasia to physicians, patients and the wider public*	* Provide information and support about euthanasia to physicians, † * Provide consultation to physicians in euthanasia requests †
	* Provide consultation to physicians in euthanasia requests * * Provide consultation to physicians in other end-of-life decisions*	

	Based on the law, when doing a consultation in a euthanasia request, the LEIF physicians has to ‡: * read the medical file * examine the patient * ascertain that the physical or psychological suffering is persistent and unbearable and cannot be relieved * make a written report of the findings	Based on the law, when doing a consultation in a euthanasia request, the SCEN physician has to § * see the patient * be convinced that the request is voluntary and well-considered * be convinced that the suffering is hopeless and unbearable * inform the patient about his/her situation and prospects * be convinced that there is no reasonable other solution * make a written report on their judgment of the due care criteria

* Source: interview with the director and training moderator of LEIF

† source: http://knmg.artsennet.nl

‡ Source: Law concerning euthanasia May 28 Wet betreffende euthanasie, 28 mei 2002. *Belgisch Staatsblad 2002 juni 2002 [Belgian official collection of the laws June 22 2002] *2002, 2002009590

§Source: Termination of Life on Request and Assisted Suicide (Review Procedures) Act April 1 Wet toetsing levensbeeindiging op verzoek en hulp bij zelfdoding 1 april, 2002.

### Functioning and organisation

SCEN or LEIF physicians must have at least five years of experience as a physician, have experience in the field of euthanasia, be skilful in consultations, and must not be a priori opposed to euthanasia as this would preclude objective consultation [20] (Table 3). Both organisations offer different training modules of roughly 23 hours given by experts, spread over several weeks, on subjects such as the performance of euthanasia, communication with patient and attending physician, and palliative care. SCEN employs actors to provide training in communication skills and lays emphasis on the consultation report, while LEIF focuses on the ethical framework of end-of-life decisions. Both SCEN and LEIF organise group meetings, called 'intervisions', where physicians can discuss concrete problems and cases with colleagues.

**Table 3:** functioning and organisation of SCEN and LEIF

Functioning & Organisation	LEIF	SCEN
Selection criteria	* 5 years experience in practice * * experience with euthanasia* * skilful in consultations* * not being fundamentally against euthanasia*	* 5 years experience in practice † * experience with euthanasia or physician-assisted death (PAD) † * skilful in consultations† * not being fundamentally against euthanasia† * write a motivation letter†

Training	24 hours spread over 5 modules in 28 weeks	22.5 hours spread over 3 days in 5 weeks
	Content of modules ‡: * general introduction * end-of-life care (laws of patient rights, palliative care and euthanasia; palliative practice) * context of the LEIF physician and the other caregivers * euthanasia in practice * communication of the LEIF physician	Content of modules § * tasks, duties and role of the consultant * communication and emotions around euthanasia * alternative possibilities and the final advice (the consultation report, suffering, palliative care)
	Intervisions ‡ * Group meetings per province * twice a year * to discuss cases	Intervisions † * Group meetings per district * 3 to 4 times a year * to air problems and to monitor the quality of consultation

Organisation	Comes under the non-profit organisation End-of-life care Academy and has 1 central secretariat for Flanders and Brussels *	Comes under a department of the Royal Dutch Medical Association and is subdivided in 23 districts throughout the Netherlands. There is a consultant network per district †

Contact	One central telephone number at LEIF secretariat, permanently available. LEIF physicians can also be contacted directly ‡	One central telephone number per district, during office hours †

Consultations	Work with guidelines received during training and use the registration form of the Federal Control and Evaluation Committee as a checklist for the criteria of due care *	Follow a written consultation protocol and have a checklist for writing the report (bron)

Expenses	No standard compensation is provided. LEIF physicians sometimes charge the price of a normal consultation *	A standard compensation of 280€ is provided via the health insurance of the patient to the SCEN physician after the SCEN physician files a report ||

Control	A guidance group, consisting of medical doctors, academics, ethics, experts in palliative care, nurses and actors, acts as a sounding board for LEIF ‡	An advice council, consisting of medical doctors, academics, a medical advisor and the project leader of SCEN, guards the objectives of the SCEN program †

Support	No more direct financial support after 2007¶	Annually 1.000.000€ support from the Dutch government ||

* Source: interview with the director and training moderator of LEIF

† Source: http://knmg.artsennet.nl

‡ Source: http://www.leif.be

§Source: overview of the KNMG training for SCEN physicians, KNMG, November 2008

|| Source: Information obtained by email from the district coordinator of SCEN

¶Source: Information obtained by email from the LEIF secretariat

There are currently 590 SCEN physicians, corresponding to one per 28000 inhabitants[27] or one per 112 physicians in the Netherlands. At first SCEN training was only offered to general practitioners but in 2007 it was also made available to specialists and nursing home physicians. Now there are 94 specialists and 53 nursing home physicians (not in table) [20] who have followed all five training modules organised by the Royal Dutch Medical Association. In Belgium, there are 161 LEIF-physicians (111 GPs and 50 specialists), i.e. one per 38270 inhabitants [28] or one per 127 physicians [29] in Flanders. These physicians have followed the minimum requirement of at least two modules (including the introductory module) (not in table).

When physicians require a SCEN or LEIF consultant, they can contact the organisations by telephone and a consultant is assigned to them. In the Netherlands there is a telephone number per SCEN district, while for Flanders there is one central number at the LEIF secretariat. However, LEIF physicians can also be contacted directly by the attending physician. After having discussed the case with the attending physician on the phone, both SCEN and LEIF physicians follow the directions as stated in the euthanasia law [4,5]. SCEN physicians can follow a consultation protocol and a checklist as a guideline. LEIF has no official consultation protocol but provides similar guidelines as SCEN during training sessions. SCEN physicians receive a standard financial compensation from the patient's health insurance company after having written a consultation report. No such compensation is provided for the LEIF physicians.

An important difference between both organisations relates to their financial support: SCEN receives €1.000.000 annually from the Dutch government, whereas the direct grant for LEIF physicians from the Belgian government was reduced from 20.000€ in 2003 to 10.000€ in 2007 and ceased in 2008. The organisation does receive some financial support for e.g. publishing the LEIF magazine, a practical guide on end-of-life decisions for the broad public [22].

## Discussion

Our study is the first to describe and compare how consultation services in cases of a euthanasia request have been established in the Netherlands and Belgium. In both countries, these consultation services are delivered by specially trained physicians who can be consulted in cases of a request for euthanasia and who offer support and information about the subject. The context in which the two organisations were founded, as well as the way they are organised and regulated, is different in each country: by providing information on all end-of-life care matters the Belgian Life End Information Forum seems to have a broader consultation role than the Dutch Support and Consultation in Euthanasia.

A methodological limitation of this study is that the description of both organisations is based on documents and therefore reflects the theoretical situation but not necessarily the situation in real terms.

One important difference between the consultation organisations in Belgium and the Netherlands is that the Belgian LEIF has a broader focus: its physicians can be consulted not only in cases of euthanasia requests but for all end-of-life issues. The context in which the legislation was developed in Belgium may account for this; in Belgium there was much more controversy than in the Netherlands and the legislature (government) wanted to put the focus on a wider range of options at the end of life. This may explain why a law optimizing the accessibility of palliative care and a law on patient rights emphasizing the right of the patient to choose the care they receive, were passed almost simultaneously with the euthanasia law [30,31]. In this context the initiators of LEIF, who have a broad background in palliative care, aimed to create a health provision not linked solely to euthanasia. The emphasis on palliative care is not so pronounced with SCEN, although SCEN physicians must consider other palliative options when doing a consultation. As the line between euthanasia and other end-of-life decisions is not always clear to attending physicians and their patients, it can be beneficial to have a service which provides consultation not only in the context of euthanasia but also concerning all medical aspects of the end of life. On the other hand, this requires consultants to have a wider area of expertise.

Another difference between the Dutch SCEN and the Belgian LEIF is that SCEN is more highly-regulated. A historical explanation for this can be found in differences in the development of the euthanasia laws and the fact that, as opposed to Belgium, euthanasia had been tolerated in the Netherlands long before the law was enacted. SCEN also has a longer history than LEIF. Another reason may be that the Royal Dutch Medical Society, which organises SCEN, is controlled and strongly supported financially by the Dutch government, whereas LEIF has no controlling body and little funding. Also a general cultural inclination to formalize practices in the Netherlands may explain why SCEN is more regulated [32].

The heavier regulation of SCEN may provide more of a guarantee that its consultations take place according to best-practice criteria. The more informal contact procedures of LEIF (e.g. that the attending physician may make direct contact with those in the network) could on the other hand have the advantage of making the service more approachable. If implemented in other countries, such a provision is probably best designed to fit in with the prevailing cultural characteristics.

Several similarities between SCEN and LEIF can be noted. Both organisations were founded to improve (the practice of) consultation in euthanasia requests by specifically training physicians for that purpose. These physicians also support and inform their colleagues on euthanasia. The amount of training time and the guidelines for consultation that are thought during this training are similar in both countries. Furthermore, both associations organise additional meetings to discuss concrete cases. SCEN as well as LEIF have a controlling board consisting of physicians, experts and academics to continuously evaluate the organisations' functioning.

Both The Netherlands and Belgium have been careful to set in place firm and substantial procedural due care requirements in order to safeguard good practice and it can be assumed that other countries intending to legalize euthanasia would do the same. However, the practical implications of legalization are not always covered by legislation. For instance, once euthanasia is legalized, what should a physician do when confronted with a request for euthanasia, and whom should they consult? The creation of specialized service for a priori consultation in euthanasia cases can play an important role. It helps physicians to relatively easily consult a competent second physician when they are confronted with a euthanasia request. Such a service may also guarantee more compliance with the due care requirements and hence function as an additional control mechanism. Research has already demonstrated such services to be of great importance to the careful performance of euthanasia [14]. For instance, the criteria for good consultation (e.g. independence from patient and attending physician, seeing the patient, writing a report) were more often met in consultations with SCEN physicians than with other physicians, and a strong relationship was found between a consultation with SCEN and notification of euthanasia [33]. It is important, however, that the physicians who are part of such services are fully trained to be able to judge the conditions for euthanasia and guarantee a good practice. Both SCEN and LEIF put emphasis on knowledge of the law and of palliative care, and on communication with the patient and the attending physician.

The evaluation report of the euthanasia law showed that SCEN physicians had been involved in 89% of all notified euthanasia cases in the Netherlands [33]. The notification reports in Belgium and a first assessment of LEIF activities [34] indicate that LEIF physicians have acted as a second physician in 54% of reported euthanasia cases in Flanders [35]. This shows the important involvement of this service in euthanasia. SCEN and LEIF can be an example for countries that have recently legalized euthanasia, like Luxembourg, or are discussing legalization. These countries can learn from the similarities and differences between both initiatives in organising such a service according to their law, health system and culture.

## Conclusion

In conclusion, this study shows that similar consultation services were developed in the Netherlands (SCEN) and in Belgium (LEIF) to provide an accessible, independent and qualified second physician in cases of a request for euthanasia. Though some important differences exist between the initiatives relating to the history and culture of the two countries, they are both intended to safeguard the practice of euthanasia. As both SCEN and LEIF play an important role in the performance of euthanasia in their respective countries, it is possible to conclude that, in countries where legislation on physician-assisted death is being considered, the development of such a service is warranted, parallel to or even incorporated into the relevant laws.

## Competing interests

The authors declare that they have no competing interests.

## Authors' contributions

YVW carried out the study by gathering documents and analyzing them and drafted and revised the manuscript. JC contributed to the design and coordination of the study, the analysis of the data and to the drafting of the manuscript. He also critically revised the manuscript. BOP, JB and LD contributed to the design of the study and critically revised the manuscript. All authors read and approved the final manuscript.

## Pre-publication history

The pre-publication history for this paper can be accessed here:

http://www.biomedcentral.com/1472-6963/9/220/prepub
